# Epilepsy With Suicide: A Bibliometrics Study and Visualization Analysis via CiteSpace

**DOI:** 10.3389/fneur.2021.823474

**Published:** 2022-01-17

**Authors:** Yi Guo, Zheng-Yan-Ran Xu, Meng-Ting Cai, Wen-Xin Gong, Chun-Hong Shen

**Affiliations:** ^1^Department of General Practice and International Medicine, School of Medicine, Second Affiliated Hospital, Zhejiang University, Hangzhou, China; ^2^Department of Neurology, Epilepsy Center, School of Medicine, Zhejiang University, Hangzhou, China

**Keywords:** epilepsy, suicide, CiteSpace, bibliometrics, visualization analysis

## Abstract

**Objective:**

The purpose of this study was to analyze the research status of epilepsy with suicide and to determine the hotspots and frontiers via CiteSpace.

**Method:**

We searched the Web of Science Core Collection (WoSCC) for studies related to epilepsy and suicide from inception to September 30, 2021. We used CiteSpace to generate online maps of collaboration between countries, institutions, and authors, and revealed hot spots and frontiers in epilepsy with suicide.

**Results:**

A total of 631 publications related to epilepsy with suicide were retrieved from the WoSCC. Andres M. Kanner was the most published author (25 papers). The USA and Columbia University were the leading country and institution in this field, with 275 and 25 papers, respectively. There were active cooperation between institutions, countries, and authors. Hot topics focused on depression, antiseizure medications, pediatric epilepsy, and risk factors of suicide in patients with epilepsy (PWEs).

**Conclusions:**

Based on the CiteSpace findings, this study detected active collaboration among countries, institutions and authors. The main current research trends include suicide caused by depression, suicide caused by the use of antiseizure medications, suicide in children with epilepsy, and risk factors for suicide in PWEs. Thus, more attention should be paid to the psychiatric comorbidity of PWEs (especially pediatric epilepsy), the suicidal tendency of PWEs, and the rational use of antiseizure medications in the future.

## Introduction

Epilepsy is a major disease burden in the world's population. The risk of suicide in patients with epilepsy (PWEs) is 2–4 times higher than that in the general population (1–3). According to a data analysis based on the American population, the suicide rate of PWEs in the United States is 22% higher than that of the general population (4). In addition, a Swedish study of PWEs found a 9-fold increase in the relative risk of suicide associated with mental illness and a 10-fold increase in the risk associated with the use of antipsychotic medications (2). Considering the heavy burden and serious consequences of suicide, it is necessary to explore the situation of suicide in PWEs, which could lead to better control and prevention of suicidal behavior in PWEs.

Bibliometrics is a method of quantitative analysis of publications by using mathematical and statistical methods (5, 6). It not only provides a basis for researchers to analyze subject hot spots and development trends and predict the developmental direction of the discipline, but also provides a reference for hospital disciplinary construction and talent training. In the present study, CiteSpace, a Java-based application, will be used for bibliometrics and visual analysis (5–10). Notably, this is the first time CiteSpace has been used for visual analysis in the field of epilepsy with suicide. Through CiteSpace, our research focused on the network of countries, institutions, and co-authors, cited reference analysis, co-occurrence keywords, keywords with citation bursts, and cluster analysis, and discussed the hot spots and trends of epilepsy with suicide.

## Methods

### Data Sources and Search Strategies

**Figure 1:**
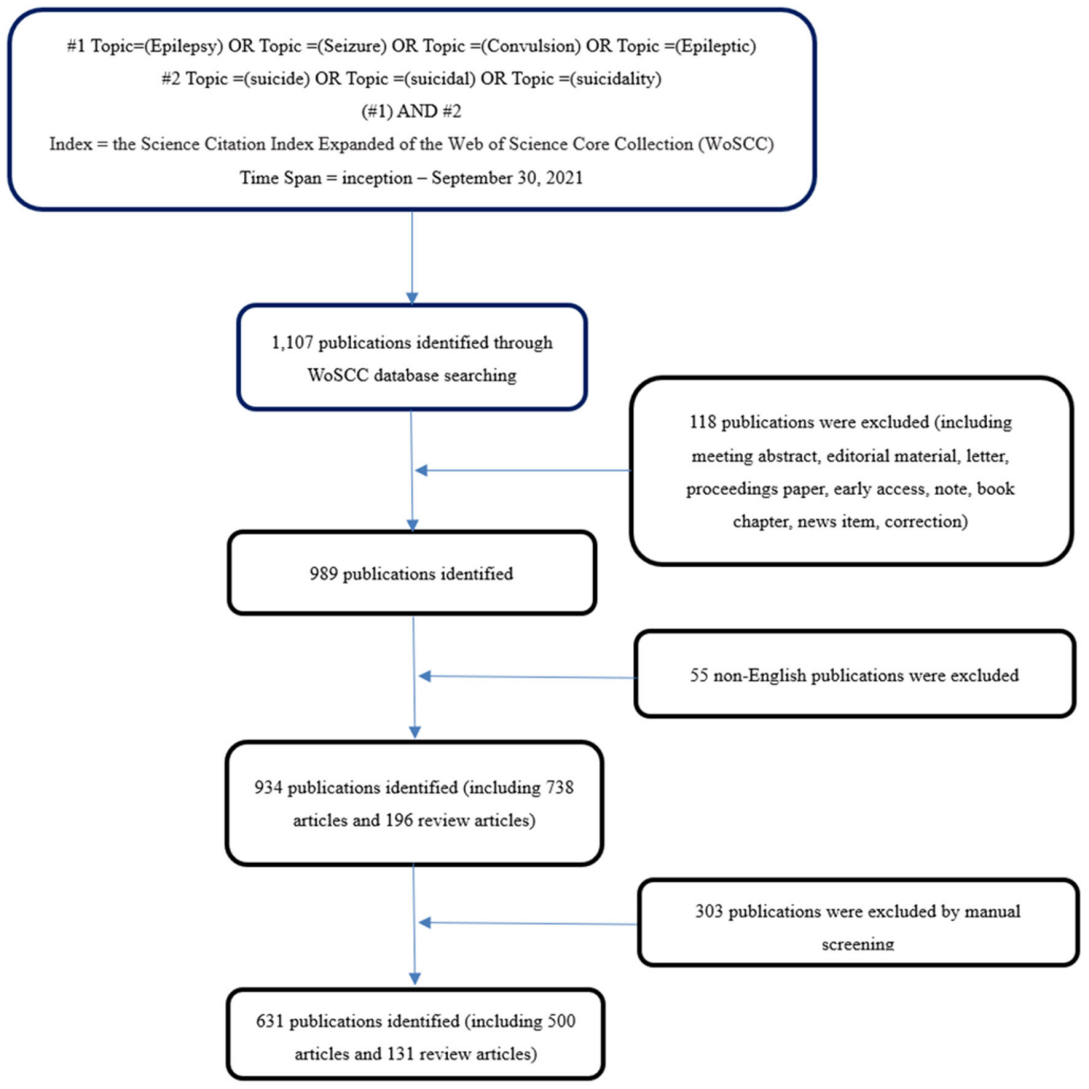
Flow chart of literature screening included in this study.

As shown in Figure 1, literature was retrieved online through the Science Citation Index-Expanded version of the Web of Science Core Collection (WoSCC). All data were acquired on December 16, 2021, so as to avoid the prejudice caused by the database update (11–13), and 1,107 results were produced.

The time span was from inception to September 30, 2021. The following terms were searched for in the Topic: (“Epilepsy” OR “Seizure” OR “Convulsion” OR “Epileptic”) AND (“suicide” OR “suicidal” OR “suicidality”) AND Language = English, only original articles and reviews were included.

### Inclusion Criteria

In light of document type, only articles and reviews were included; and the language was restricted to English only. Referring to other studies (13, 14), two independent investigators reviewed the titles and abstracts and deleted studies that were not associated with epilepsy and suicide. After reviewing the titles and abstracts, 631 publications were remained.

### Visualization Analysis Tool—CiteSpace

CiteSpace identified research frontiers and emerging trends in the field of epilepsy with suicide by analyzing the trend of the annual number of publications and growth, exploring collaboration networks between authors/institutions/countries, identifying co-cited references, as well as capturing keywords with strong citation bursts over time. There are different nodes and links in various CiteSpace visualization knowledge graphs, and the nodes with high centrality are usually identified as hot spots or turning points in this domain. We downloaded the records retrieved by WoSCC, and then converted these data into plain text format for export, including complete records and references, which was named download_ XXX. txt, and finally imported into citespace.5.7.R2W for bibliometric and visual analysis. Cluster analysis of co-occurrence keywords revealing the main topics was performed using CiteSpace. The silhouette function is usually used to evaluate the clusters. Generally speaking, if the silhouette value is over 0.7, it means that the members of the cluster have high homogeneity, indicating that the clustering result is meaningful. If it is >0.5, clustering is generally considered reasonable.

## Results

### Bibliometric Analysis of Publication Years

**Figure 2:**
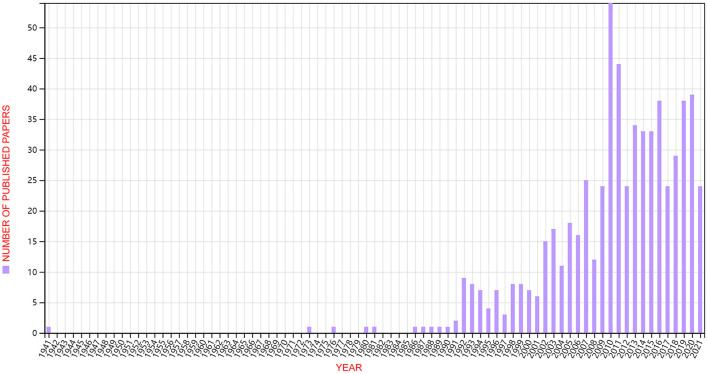
Annual trend chart of publications. The time span was from inception to August 31, 2021.

A total of 631 publications were downloaded from the WoSCC database (Figure 1). As shown in Figure 2, the number trend of annual publications related to epilepsy combined with suicide showed fluctuating growth, from one publication in 1941 to 39 publications in 2020, and 24 publications from January 1, 2021 to September 30, 2021. In particular, there was rapid growth in the 2010s, which indicated that this field received increased attention in the 2010s.

### Bibliometric Analysis of Countries and Institutions

**Figure 3:**
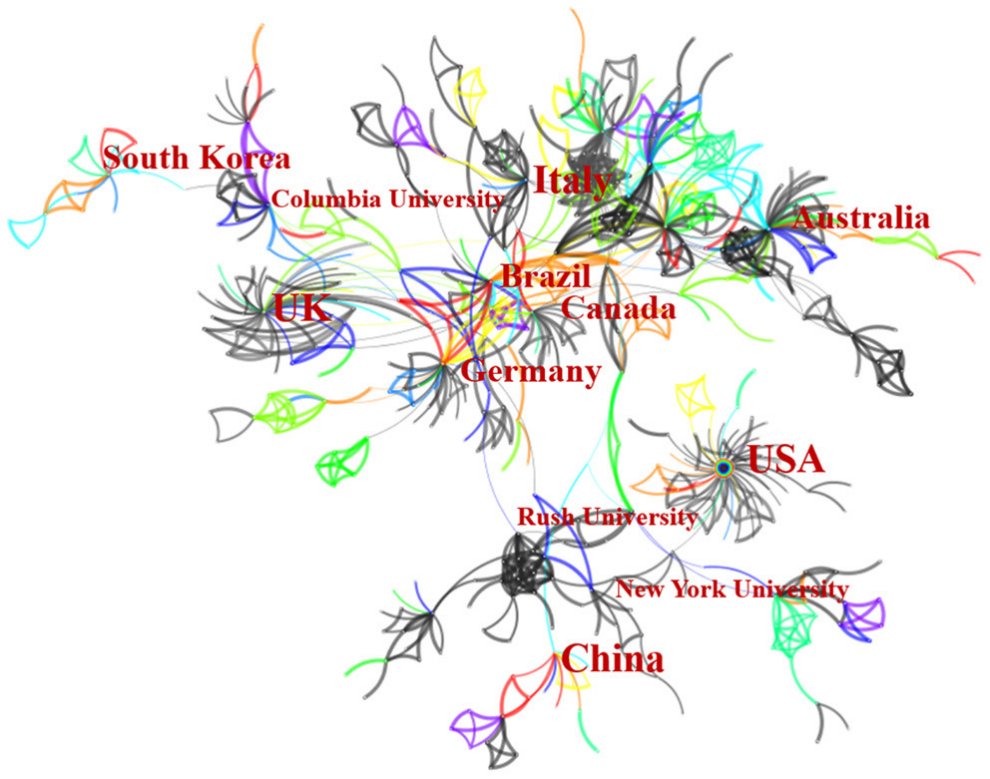
The network of countries and institutions.

The top nine countries and the top five institutions contributed 586 articles (92.87%) and 77 articles (12.20%), respectively (Table 1). The top five countries were the USA, UK, Germany, Canada, and Italy. The top five institutions were Columbia University, Rush University, New York University, Emory University and Harvard University. The results showed active cooperation among institutions and countries, especially in the United States and European countries (Figure 3).

**Table 1:** The top 9 countries and top five institutions of epilepsy with suicide.

**Rank**	**Country**	***N*** **(%)**	**Institution**	***N*** **(%)**
1	USA	275 (43.58)	Columbia University	25 (3.96)
2	UK	98 (15.53)	Rush University	16 (2.54)
3	Germany	50 (7.92)	New York University	16 (2.54)
4	Canada	34 (5.39)	Emory University	10 (1.58)
5	Italy	31 (4.91)	Harvard University	10 (1.58)
6	China	26 (4.12)		
7	Australia	26 (4.12)		
8	Brazil	23 (3.65)		
9	South Korea	23 (3.65)		

### Bibliometric Analysis of Co-authors

**Figure 4:**
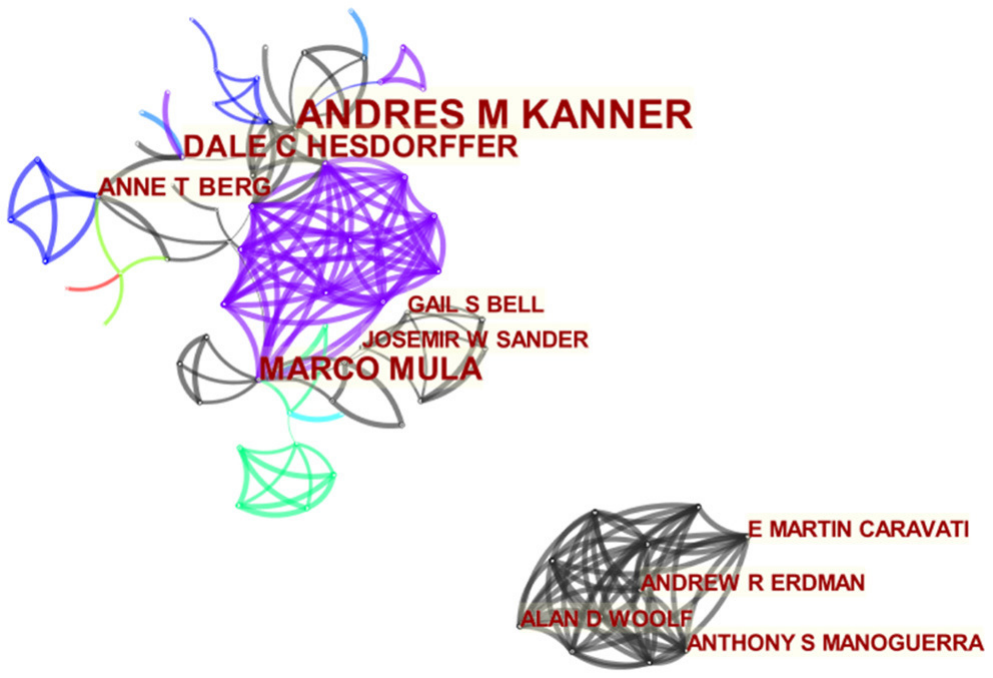
The network of co-authors.

Figure 4 is the network of co-author generated by CiteSpace. Different nodes represent different authors, and the size of the circle represents the number of publications of different authors. Larger nodes mean more releases. The top 10 authors contributed 84 papers (13.31%) (Table 2). The most prolific authors were Andres M. Kanner (25, 3.96%), followed by Marco Mula (12, 1.90%), Dale C. Hesdorffer (11, 1.74%), and Anne T. Berg (6, 0.95%). The thicker the line between different circles, the more collaboration between authors, and as shown in Figure 4, productive authors usually had stable collaborations with other authors.

**Table 2:** The top 10 authors of epilepsy with suicide.

**Rank**	**Author**	**Count of articles**	**Year of first article**
1	Andres M. Kanner	25	2000
2	Marco Mula	12	2009
3	Dale C. Hesdorffer	11	2007
4	Anne T. Berg	6	2010
5	Alan D. Woolf	5	2006
6	Andrew R. Erdman	5	2006
7	Anthony S. Manoguerra	5	2006
8	E. Martin Caravati	5	2006
9	Gail S. Bell	5	2009
10	Josemir W. Sander	5	2009

### Bibliometric Analysis of Co-cited References

Table 3 shows the top 10 studies related to epilepsy with suicide, which were cited over 300 times. The first co-cited reference was a paper published by Christensen et al. (3), which proved that PWEs had a higher risk of suicide, even if there were differences in mental diseases, demographic differences, and socio-economic factors at the same time. The second co-cited reference was published by Hesdorffer et al. (15), which recommended routine evaluation of depression, anxiety, and suicidal tendencies in all PWEs, and stated that future clinical trials should be carried out including effective instrumental systems to assess these conditions to determine whether the possible signals observed by the US Food and Drug Administration (FDA) are genuine. The third co-cited reference was also published by Hesdorffer et al. (16), which confirmed that major depression was a risk factor for unprovoked seizure. However, the relationship between depression and unprovoked seizure is more complex than previously thought: severe depression and attempted suicide are independent risk factors for the development of unprovoked seizure. Obviously, patients with new unprovoked seizure should evaluate their history of suicide attempt and severe depression, which will help to choose treatment and prevent suicide.

**Table 3:** The top 10 co-cited references sorted by the number of citations.

**Rank**	**Co-cited reference**	**Count**
1	Christensen et al. (3)	46
2	Hesdorffer et al. (15)	32
3	Hesdorffer et al. (16)	31
4	Arana et al. (17)	31
5	Tellez-Zenteno et al. (18)	31
6	Hesdorffer et al. (19)	29
7	Andersohn et al. (20)	28
8	Patorno et al. (21)	28
9	Bell et al. (1)	27
10	Olesen et al. (14)	24

### Bibliometric Analysis of Co-occurring Keywords and Cluster

**Figure 5:**
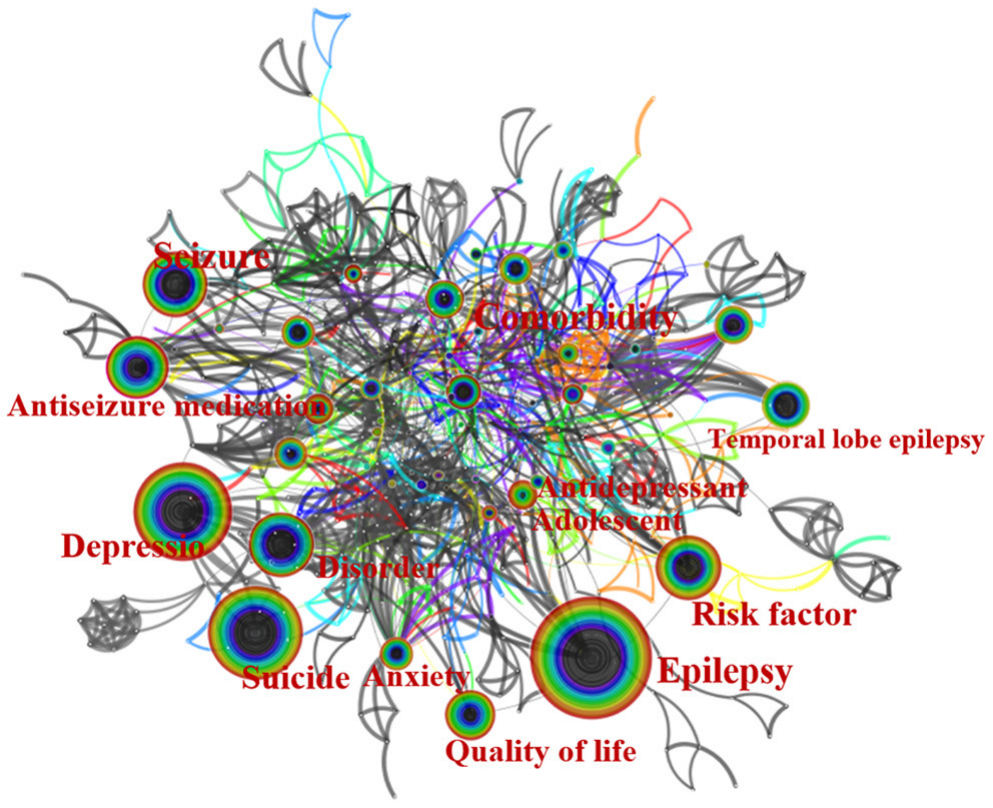
The network map of keywords.

High central keywords reflect the status and influence of the corresponding research content in the research field, while high-frequency keywords represent a hot topic in a research field. As shown in Table 4, the top 10 high-centrality keywords were: Antidepressant (centrality: 0.24), Antiseizure medication (centrality: 0.18), Anticonvulsant (centrality: 0.18), Anxiety (centrality: 0.16), Carbamazepine (centrality: 0.15), Bipolar disorder (centrality: 0.13), Anxiety disorder (centrality: 0.13), Children (centrality: 0.12), Adolescent (centrality: 0.12), and 5-HT1A receptor binding (centrality: 0.12). The top ten high-frequency keywords were Epilepsy (frequency: 275), Depression (frequency: 217), Suicide (frequency: 185), Antiseizure medication (frequency:113), Disorder (frequency: 112), Risk factor (frequency: 112), Seizure (frequency: 108), Quality of life (frequency: 89), Temporal lobe epilepsy (frequency: 85), and Comorbidity (frequency: 62). In addition, Figure 5 shows the keywords network.

**Table 4:** The top 10 keywords of epilepsy with suicide.

**Rank**	**Count**	**Keywords**	**Centrality**	**Keywords**
1	275	Epilepsy	0.24	Antidepressant
2	217	Depression	0.18	Antiseizure medication
3	185	Suicide	0.18	Anticonvulsant
4	113	Antiseizure medication	0.16	Anxiety
5	112	Disorder	0.15	Carbamazepine
6	112	Risk factor	0.13	Bipolar disorder
7	108	Seizure	0.13	Anxiety disorder
8	89	Quality of life	0.12	Children
9	85	Temporal lobe epilepsy	0.12	Adolescent
10	62	Comorbidity	0.12	5-HT1A receptor binding

**Figure 6:**
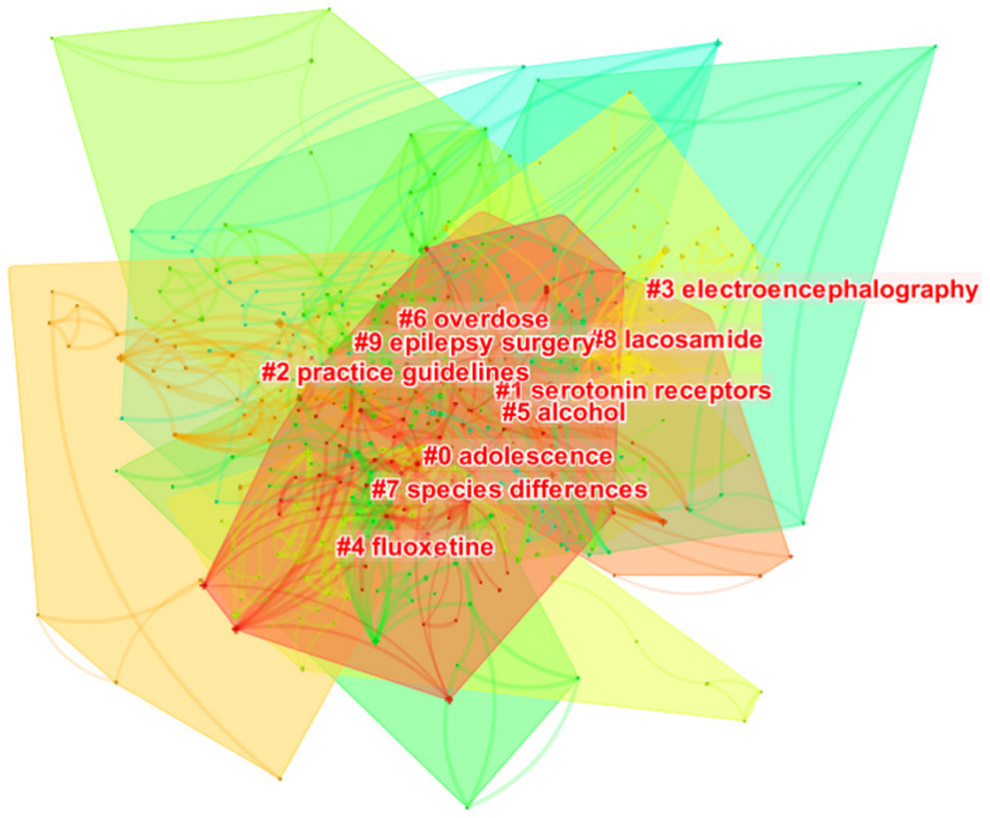
Keywords cluster analysis co-occurrence map.

Ten clusters were obtained, and the silhouette value of each cluster was above 0.8, manifesting that the results were credible and significant (Figure 6, Table 5).

**Table 5:** Keywords cluster analysis.

**Cluster ID**	**Silhouette**	**Label (LLR)**	**Included keywords (top 5)**	**Mean (Year)**
0	0.885	Adolescence	Disorder; quality of life; risk; adolescent; behavior	2005
1	0.819	Serotonin receptors	Prevalence; major depression; psychiatric comorbidity; suicidality; psychiatric disorder	2012
2	0.924	Practice guidelines	Antiseizure medication; children; double blind; complex partial seizure; Alzheimer's disease	2004
3	0.917	Electroencephalography	Temporal lobe epilepsy; suicidal ideation; anticonvulsant; surgery; mood	2006
4	0.894	Fluoxetine	Depression; suicide; antidepressant; fluoxetine; multiple sclerosis	2000
5	0.930	Alcohol	Epidemiology; health; validation; inventory; injury	2007
6	0.855	Overdose	Therapy; carbamazepine; overdose; topiramate; toxicity	2000
7	0.894	Species differences	Anxiety; efficacy; adverse event; pediatric epilepsy; predictor	2007
8	0.914	Lacosamide	Suicide attempt; safety; levetiracetam; adverse effect; partial onset seizure	2012
9	0.805	Epilepsy surgery	Seizure; people; population; bipolar disorder; symptom	2007

### Bibliometric Analysis of Keywords With Citation Bursts

**Figure 7:**
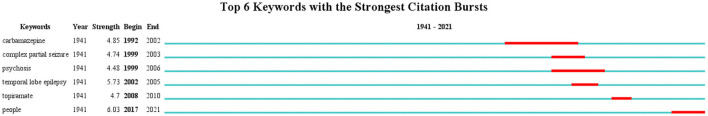
Top 6 keywords with the strongest citation bursts.

Figure 7 reflected the research frontier and showed the top 6 keywords with the strongest citation bursts. The red line represents the time period of the keyword burst, and the blue line indicates the time interval. The top 6 keywords with the citation bursts first appeared in 1941 (carbamazepine, complex partial seizure, psychosis, temporal lobe epilepsy, topiramate, people). In addition, one keyword (people) with the citation bursts persisted until 2021, indicating that the research frontier is people.

## Discussion

### General Information

This is the first study to review the research status of epilepsy with suicide through CiteSpace and to reveal the associated research hotspots and frontiers. A total of 631 publications related to epilepsy with suicide were acquired from the WoSCC, from inception to September 30, 2021, and their growth trend fluctuated over time. The top 9 countries engaged in the study of epilepsy with suicide contributed 586 articles, accounting for 92.87% of the total number of publications. The USA contributed 275 articles (about one-third of the total number of publications), reflecting its dominant position in the study of epilepsy with suicide. As one of the two developing countries in the top 9, China has made great achievements in epilepsy with suicide in the past. However, the attention paid to PWEs with suicidal symptoms is still insufficient in China, especially in Southwest China, a relatively underdeveloped area (22). Suicide is a cause of premature death in PWEs, and the rate of death by suicide in PWEs is 2.06–4.6 times that of the general population; therefore, China needs to pay more attention to this aspect in the future, especially the suicide of PWEs in Southwest China (22).

In terms of the number of publications, institutions with strong scientific research strength were mainly concentrated in higher education research institutions. The top 5 institutions published 77 articles, accounting for 12.20% of all articles. These five institutions are all located in the USA, indicating that American institutions rank first in light of absolute contribution and relative influence, which is accordance with the analysis of the contribution of countries in the field of epilepsy with suicide. The close relationship between the level of health care and the speed of economic development, mean that the institution distribution table provides valuable information to help researchers identify and select appropriate cooperative institutions.

The top 10 authors published 84 articles, accounting for 13.31% of all articles. Although Andres M. Kanner was the most prolific author (25 articles), the high-frequency cited literature did not come from him. This shows that the author needs to strengthen cooperation with other scientific research institutions, promote progress in relevant fields, and improve the quality of the articles.

### Hot Issues in Epilepsy With Suicide Research

Keywords represent the high-level summary and conciseness of the topic. In the process of analysis, frequently-used keywords are usually used to recognize hot spots in the research field. The results of co-occurrence keywords and cluster analysis indicated that main current research trends include suicide caused by depression, suicide caused by the use of antiseizure medications, suicide in children with epilepsy, and risk factors for suicide in PWEs.

#### Depression

Depression is an under-diagnosed and under-treated comorbidity in children and adults with epilepsy (23, 24). Depression was common in PWEs, and a systematic review and meta-analysis showed that 23.1% of PWEs suffered from depression, which was five times higher than that of the general population (24, 25). In addition, mental disorders, especially depression and anxiety, were associated with suicidal tendencies in PWEs (4, 26–28). Chia-hung Kao et al. found that epilepsy accompanied by depression increased the risk of suicide attempts and suicidal drug overdose in Chinese Taiwan patients, and female patients younger than 65 years old had a further increased risk of suicide attempts in PWEs and depression (29). More recently, Hesdorffer et al. showed that attempted suicide increases the risk of epileptic seizures by 5.1-fold, and that both depression and attempted suicide are important and independent risk factors for the development of epilepsy (16, 30). The risk of suicide in PWEs is 1.2–5 times higher than that of the general population (31). Epilepsy, depression, and suicidal behavior have been shown to share a common pathogenic mechanism in their etiology. Using a comprehensive database of all suicides in northern Finland between 1988 and 2002 (*n* = 1,877), as well as information on physical and psychiatric disorders treated in all hospitals, Mainio et al. assessed the relationship between epilepsy, suicidal behavior, and depression, and found that 1.3% of the victims suffered from hospital-treated epilepsy. Compared with other suicide victims, epilepsy patients were more likely to be female, older, and were more likely to suffer from depression (32). It is recommended to conduct routine and regular screening for all PWEs, to use reliable screening tools such as Neurological Disorders Depression Inventory for Epilepsy (NDDI-E) (23, 33, 34), and to identify patients in need of help in a timely manner. Neurologists should be able to manage mild to moderate depression; however, for severe and difficult to treat depression, or for patients with serious suicidal tendencies, a referral to a mental health specialist must be made (34, 35). Therefore, it is important to manage and treat depression in PWEs, and improving specialist care, epilepsy control, and patient education might be the most important measures to reduce seizure-related mortality.

#### Antiseizure Medications

In January 2008, the US Food and Drug Administration (FDA) issued a general warning on the possible increased risk of suicide associated with ASMs, which caused concern and some confusion. It now seems clear that the FDA's warning was based on data affected by some methodological limitations (36). Some studies revealed that the use of ASMs was not related to an increased risk of suicide-related events in PWEs, but was associated with an increased risk of suicide-related events in patients with depression and non-epileptic patients (32, 37–39). Among neurologists who treat PWEs, only 8% said they would provide patients with written information about suicide risk, and the vast majority said they would not change their routine clinical practice (40). Some studies have shown that carbamazepine and sodium valproate have anti-suicide effects on PWEs, while lamotrigine, oxcarbazepine, and gabapentin might prevent suicide (2, 41–45). By contrast, other studies (46, 47) have found that the total risk of suicide increased two to four times after using ASM; clonazepam, sodium valproate and phenobarbital might double the risk of suicide in a short time after the start of treatment, among which lamotrigine had the highest risk (46); activation of suicidal ideation increased with rufinamide (48); and the use of new ASMs, including levetiracetam, topiramate, and vigabatrin, led to a 3-fold increase in the risk of suicide-related behavior when compared with no ASM use (20, 22, 47). The use of gabapentin, oxcarbazepine, tiagabine, lacosamide, and phenytoin was associated with an increased risk of suicide or violent death compared with the use of topiramate (21, 41, 49, 50). The reasons leading to the contradictions among research results might be related to the history of suicidal behavior, familial tendency mental disorder, and comorbidity of mental diseases. One study found that the suicide risk of ASM users with suicidal behavior history and familial tendency mental disorder was much higher than that of people without suicidal behavior history and familial tendency mental disorder (15, 44). So far, it is unclear whether ASM therapy affects the risk of suicide, but the risk of stopping ASMs or refusing to initiate ASMs is significantly greater and might result in serious harm, including death of the patient. Therefore, it is suggested that we should be cautious and classify the impact of medications on suicide. In the future, further research should be conducted on the relationship between ASMs and suicide needs. Special attention should be paid to psychiatric comorbidity and suicidal thoughts, to clarify the role of ASMs in suicidal behavior and distinguish drug effects from disease-related effects.

#### Pediatric Epilepsy

Studies on suicidal behavior in adult epilepsy patients are more frequent; however, few studies have examined suicidal behavior in children with epilepsy (1). Caplan et al. (51) reported that 20% of the samples of Childhood Absence Epilepsy (CAE) or Complex Partial Seizure (CPS) in children aged 5 to 16 had suicidal ideation, and 37% of the people with suicidal ideation had suicidal plans, but no suicidal attempts. Children with both disruptive and affective / anxiety disorders were 12 times more likely to agree with the idea of suicide. Tatiana Falcone et al. found a significant result that 11% of pediatric epilepsy patients without any confirmed psychiatric diagnosis reported suicidal thoughts on the self-report questionnaire (52). Jones et al. stressed that according to psychiatric interviews, children with epilepsy were at high risk of suicidal ideation if they had more than one psychiatric diagnosis. When the number of psychiatric diagnoses was examined, each additional diagnosis increased the likelihood of suicidal ideation by 80% (53). In addition, Jones et al. (31) showed that it was essential to provide assistance and intervention or treatment in the first year of suicidal behavior symptoms. So far, little is known about the initial manifestations of epilepsy suicide in children. However, Jones provided evidence that a quarter of children with chronic epilepsy had a sensitive predictor of suicidal ideation, in which the Child Behavior Checklist (CBCL) total behavior problems scale was a useful tool to detect suicidal ideation in children with epilepsy (53). In a word, suicide is a very serious social problem, and identifying and preventing suicide at a younger age is the focus of our future research.

#### Risk Factors

Jones et al. concluded that hospitalized children with a history of mental disease, low socio-economic status, and an unstable somatic disease were the main risk factors for suicide attempts (53). Seo et al. (54) found that the history of febrile convulsion was a risk factor for suicide. A nested case-control study was designed in a group of PWEs, suggesting that unemployment, levetiracetam use, depression, and stigma are independent risk factors for suicide in Chinese PWEs (22). In addition, suicide risk factors for epilepsy also included high antiseizure medication load, frequent seizures, family relationship, family history, health problems, severe anxiety, and access to guns or other lethal methods, physical or sexual abuse, medical abortion, and cognitive impairment (16, 31, 42, 55–59). These risk factors can be managed by educating patients and nursing staff to understand the neuropsychiatric symptoms of epilepsy, avoiding or carefully monitoring ASMs with a relatively high risk of neuropsychiatric complications in patients who may have the tendency of psychiatric comorbidity, and timely psychiatric referral (37). Therefore, it is recommended that all physicians involved in the treatment of epilepsy make a psychiatric assessment to judge the possible suicide risk factors. In terms of future research direction, it is unique or common for PWEs to better understand the nature and mortality of suicide attempts, as well as the risk factors of suicide attempts and suicide.

### Strengths and Limitations

To the best of our knowledge, this is the first time CiteSpace has been used to conduct bibliometric analysis and for the visual display of epilepsy with suicide in terms of hot spots, co-cited literature, and collaborations between countries, institutions and authors. There was no time limit for our literature search. The data downloaded from the WoSCC database covered the vast majority of articles in the field of epilepsy with suicide research, and the data analysis was relatively objective and comprehensive, which clarified the past and present situation of epilepsy with suicide and predicted future research frontiers. However, this study also had some limitations. Firstly, the limitations of CiteSpace meant that we only downloaded literature analysis from WoSCC; therefore, our data might not represent all the available literature. Secondly, our study defined certain key words that may lead to data reduction. Thirdly, our analysis only included articles in English, which made the analysis incomplete to some extent. Finally, because of the existence of multiple synonyms, there might be some overlap between different categories of content in the keyword clustering.

## Conclusion

Based on the findings of CiteSpace findings, this study detected positive collaboration among countries, institutions, and authors. Main current research trends include suicide caused by depression, suicide caused by the use of antiseizure medications, suicide in children with epilepsy, and risk factors for suicide in PWEs. Thus, more attention should be paid to the psychiatric comorbidity of PWEs (especially pediatric epilepsy), the suicidal tendency of PWEs, and the rational use of antiseizure medications in the future.

## Data Availability Statement

The raw data supporting the conclusions of this article will be made available by the authors, without undue reservation.

## Author Contributions

YG designed and analyzed the data. YG and Z-Y-RX drafted and edited the manuscript. YG, C-HS, M-TC, and W-XG contributed to revising the manuscript. All authors contributed to the article and approved the submitted version.

## Funding

This study was supported by the National Natural Science Foundation of China (Grant No. 81871010).

## Conflict of Interest

The authors declare that the research was conducted in the absence of any commercial or financial relationships that could be construed as a potential conflict of interest.

## Publisher's Note

All claims expressed in this article are solely those of the authors and do not necessarily represent those of their affiliated organizations, or those of the publisher, the editors and the reviewers. Any product that may be evaluated in this article, or claim that may be made by its manufacturer, is not guaranteed or endorsed by the publisher.
